# Antioxidant N-Acetylcysteine Attenuates Hepatocarcinogenesis by Inhibiting ROS/ER Stress in TLR2 Deficient Mouse

**DOI:** 10.1371/journal.pone.0074130

**Published:** 2013-10-02

**Authors:** Heng Lin, Xiao-bo Liu, Jiao-jiao Yu, Fang Hua, Zhuo-wei Hu

**Affiliations:** Molecular Immunology and Pharmacology Group, State Key Laboratory of Bioactive Substance and Function of Natural Medicines, Beijing Key Laboratory of New Drug Mechanisms and Pharmacological Evaluation Study (NO. BZ0150), Institute of Materia Medica, Peking Union Medical College and Chinese Academy of Medical Sciences, Beijing, China; Enzo Life Sciences, Inc., United States of America

## Abstract

Hepatocellular carcinoma (HCC) remains one of the most deadly solid tumor malignancies worldwide. We recently find that the loss of toll-like receptor 2 (TLR2) activities promotes the diethylnitrosamine (DEN) induced hepatocellular carcinogenesis and tumor progression, which associates with an abundant accumulation of reactive oxygen species (ROS) and endoplasmic reticulum (ER) stress. This finding suggests that the ROS/ER stress plays a role in TLR2 modulated carcinogenesis of HCC. To investigate the mechanism of TLR2 activity defending against hepatocarcinogenesis, the TLR2-deficient mice were treated with or without antioxidant N-acetylcysteine (NAC) before DEN administration. We found that pretreatment of these animals with NAC attenuated carcinogenesis and progression of HCC in the TLR2-deficient mice, declined ROS/ER stress, and alleviated the unfold protein response and inflammatory response in TLR2-deficient liver tissue. Moreover, the NAC treatment significantly reduced the enhanced aggregation of p62 and Mallory-Denk bodies in the DEN-induced HCC liver tissue, suggesting that NAC treatment improves the suppressive autophagic flux in the TLR2-deficient liver. These findings indicate that TLR2 activity defends against hepatocarcinogenesis through diminishing the accumulation of ROS and alleviating ER stress and unfold protein response mediated inflammatory response in the liver.

## Introduction

Hepatocellular carcinoma (HCC) is one of the most commonly diagnosed solid tumor malignancies and ranks as the third leading cause of cancer-related death worldwide [1], [2]. A verity of the risk factors, including hepatitis virus infection, long-term excessive alcohol intake, and nonalcoholic steatohepatitis elicit the chronic-inflammatory response to promote the initiation and progression of HCC. Although the cellular transformation of HCC is known to be a multistage process starting with increased cell density, followed by atypia and frequent stromal invasion [3]–[5], the underlying molecular mechanisms of hepatocarcinogenesis remain to be completely elucidated [6], [7].

Toll-like receptors (TLRs) are a family of pattern recognition receptors (PRRs) that regulate the innate immune response [8]. They recognize distinct pathogen-associated molecular patterns (PAMPs) [9], [10], such as fractions of bacteria, virus, or fungi, and damage-associated molecular patterns (DAMPs) released from the damaged tissues. The activation of TLRs triggers immune responses and builds up the first barrier against foreign invasion or cellular malignance transformation. However, the activation of TLRs may also lead to an uncontrolled and up-regulated innate immune response, which in turn may result in inflammatory liver disorders and HCC [11]. TLRs are expressed in human liver, where they become activated upon exposure to intestinal bacteria which translocate via the portal vein [12]–[14]. Almost all types of liver cells, including hepatocytes [15], kupffer, stellate and sinusoidal endothelial cells, express the functional TLR2 in the liver [16]. In primary hepatocytes, TLR2 ligands can activate the nuclear factor kappa B (NF-κB) pathway [15]. Human genetic studies have revealed a significant association between TLR2 polymorphisms and HCC susceptibility [17], [18]. TLR2/4 activation was implicated in the subsequent tumorigenesis in mouse model of liver injury induced by 3,5-diethoxycarbonyl-1,4-dihydrocollidine (DDC) [19]. In contrast, mice deficient in TLR4 showed a marked increase [20], [21], while MyD88 deficient mice showed a marked decrease in DEN-induced liver tumors [11]. Using the same well-established mouse HCC model, we find recently that the TLR2 signaling plays a defense role against HCC, through eliciting intracellular senescence and maintaining autophagy flux in liver cells [22], [23].

Oxidative stress, which results from the generation of ROS by environmental factors or mitochondrial dysfunction, associates with liver tumorigenesis for either the direct causation of DNA mutation or the causal link with the chronic inflammation [24], [25]. Oxidative stress can be enforced by endoplasmic reticulum (ER) stress, autophagic flux failure, or the accumulation of p62 aggregates [26]–[28]. Meanwhile, cells undergoing high oxidative stress can be cleared by the p38-MAPK-NFκB pathway mediated apoptosis [29]. It is possible that these signaling factors and intracellular processes may be interrelated and/or triggered by a common upstream factor that is causative of or responsive to transformation of HCC.

In current study, the ROS and ER stress were found abundantly accumulated in the DEN-induced HCC in TLR2-deficient mice. Therefore, we investigated whether ROS is directly responsible for the aggravated HCC because of TLR2 deficiency. We found that the pretreatment of TLR2 deficient mice with antioxidant NAC had rescued the development and progression of HCC in these animals. Our studies indicate that, at least partially, by limiting ROS accumulation and ER stress, TLR2 signaling plays a critical role in defending against hepatocarcinogenesis.

## Experimental Procedures

### DEN-induced liver cancer model

All procedures involving mice were carried out with prior approval from the Animal Care and Use Committee of Institute of Materia Medica, China. TLR2-deficient mice with B6 background and WT B6 mice (all males) were obtained from Jackson Laboratory (Bar Harbor, ME, USA) and housed in pathogen-free conditions. At 14 days old, a subset of WT mice were selected for pre-treatment with anti-TLR2 antibody (to block TLR2 signaling), IgG (negative control), and a subset of TLR2^−/−^ mice were selected for pre-treatment with NAC (antioxidant) or physiological saline (n = 15 to 20 per group). At 15 days old, all of the pre-treated and untreated WT and untreated TLR2^−/−^ mice were injected with 25 mg/kg DEN (Sigma-Aldrich, St. Louis, MO, USA) to induce HCC. Over the next six months, the anti-TLR2- and IgG-pretreated mice received weekly boosters (100 µg/kg). The NAC-pretreated mice received every-other-day boosters (100 mg/kg) for only three months and were left untreated for the remaining three months. At the end of the six months, all mice in the five groups were sacrificed to evaluate the development of liver cancer. Tumor number and size were recorded. For this, the whole liver was excised from each animal and washed in cold PBS. An investigator who was blinded to the animal's identity counted the numbers of surface liver tumor nodules (>0.5 mm) for all liver lobes. After counting, the livers were divided, with one portion being stored in cold PBS and the other being sectioned and fixed in 10% formalin for histological analysis using hematoxylin-eosin (H&E) staining. To investigate the mechanism of carcinogenesis, mice in WT, TLR2^−/−^, physiological saline-pretreated TLR2^−/−^, NAC-pretreated TLR2^−/−^ groups were sacrificed at the end of the one months after DEN treatment for western blot analysis (n = 4 per group).

### Cells, plasmid and transfection

Liver parenchymal and non-parenchymal cells were isolated and plated as previously described [30]. Parenchymal cells were treated with or without 50 µM of H_2_O_2_ for 2 hrs and were accessed for ROS related p62 aggregates measurement (n = 4 per group). Mouse embryonic fibroblasts (MEFs) were primary isolated and plated as previously described [31]. MEFs were treated with or without DEN (200 µg/ml) for 24 hr, and were harvested for western blot measurement (n = 4 per group). TLR2 expressing plasmid pDUO-CD14/TLR2 was purchased from InvivoGen (San Diego, CA). HepG2 cell line was obtained from the ATCC (Rockville, MD, USA). To generate HepG2 cell populations stably expressing TLR2, the control and the TLR2 expressing plasmids were both transfected into HepG2 cells with Lipofectamine2000 (Invitrogen, Carlsbad, CA, USA) according to the manufacturer's instructions. After 24 hours of transfection, stable transfectants were selected in medium containing 1 µg/ml puromycin (InvivoGen, San Diego, CA). Single clones were amplified by a dilution cloning technique.

### Preparation of Triton X-100-insoluble aggregates and p62 analysis

Liver tissues were gently homogenated and lysed in RIPA lysis buffer for 15 min on ice. The fraction of Triton X-100-soluble proteins was collected by centrifugation (13000 rpm, 4°C, 15 min). The insoluble pellet was washed with RIPA buffer, re-solubilized by incubating with RIPA buffer containing 2 M urea, and collected by centrifugation. After separation through SDS-Gel, Triton X-100-soluble and insoluble p62 were detected by immune blotting (n = 4 per group).

### Western blot analysis

Liver tissues (n = 4 per group) were gently homogenated and lysed in RIPA lysis buffer for 15 min on ice. Total protein was collected by centrifugation (13000 rpm, 4°C, 15 min) and quantified using a bicinchoninic acid kit. Equal amounts of protein from each mouse liver were loaded onto a SDS-Gel, resolved by electrophoresis, and transferred to a PVDF membrane. Non-specific binding was blocked by incubating with 5% nonfat milk solution. 8-ohdG, p62, Bip, phospho-eIF2α, eIF2α, CHOP, IRE1α, phospho-JNK1/2, JNK1/2, and cleaved caspase-3 were detected by incubation with the corresponding primary antibodies, including anti-8-ohdG (Santa Cruz Biotechnology, CA, USA), anti-p62 (Sigma-Aldrich, St. Louis, MO, USA), anti-Bip, anti-phospho-eIF2α, anti-eIF2α, anti-CHOP, anti-IRE1α, anti-phospho-JNK1/2, anti-JNK1/2, and anti-cleaved caspase-3 (Cell Signaling Technology, MA, USA), followed by incubation with the appropriate enzyme-conjugated secondary antibodies (Invitrogen, CA, USA). The immunoreactive bands were visualized with chemiluminescence using the ECL Western Blotting Substrate (Pierce) and following the manufacturer's protocol.

### ROS assay

The molecular probe, 2′,7′-Dichlorofluorescein Diacetate (H2DCF-DA, Sigma-Aldrich, St. Louis, MO, USA) was used to detect ROS. Liver cells were in situ released by enzymatic digestion with collagenase, and were loaded with H2DCF-DA (10 µM in phenol-free medium) for 30 min (n = 4 per group). Cells were then rinsed with PBS and measured by flow cytometer (Becton-Dickinson Biosciences) using the accompanying CellQuest software. For each measurement, >10,000 events were counted.

Frozen liver sections (n = 4 per group) were incubated with 5 µM Dihydroethidium (DHE, Sigma-Aldrich, St. Louis, MO) for 30 min (37°C). After washes with PBS, the ROS level in the tissue was measured by confocal microscopy (Leica).

### ROS related P62 aggregates assay

Liver parenchymal and non-parenchymal cells were primary isolated and were loaded with 5 µM DHE for 30 min (37°C, n = 4 per group). Cells were rinsed with PBS containing 0.1% saponin, blocked with 1% BSA, and incubated with antibodies against p62 for 30 min (25°C). Cells were then washed with PBS, incubated with donkey anti-rabbit IgG labeled with Alexa Fluor 488 (Invitrogen, CA, USA) at 4°C for 30 min, and measured by flow cytometer (Becton-Dickinson Biosciences) using the accompanying CellQuest software. For each measurement, >10,000 events were counted.

### Immunohistochemistry and immunofluorescence

A portion of the unprocessed liver tissues were fixed in 4% paraformaldehyde, dehydrated, embedded in paraffin, and sliced into 4 µm thick sections. After dewaxing in xylene and rehydrating in a graded alcohol series, the sections were processed for antigen retrieval by heating in citrate buffer, pH 6.0, for 30 min at 95°C. Immunodetection of TLR2 and p62 was carried out in a humidified chamber, as follows (n = 4 per group). After three washes in PBS for 5 min each, the sections were covered with 3% bovine serum albumin (BSA) and incubated at 37°C for 30 min. Then, primary antibody replaced the BSA and the slides were incubated at 4°C overnight. After three washes in PBS for 5 min each, the sections were incubated with goat anti-rabbit IgG labeled with horseradish peroxidase-streptavidin complex (Invitrogen, CA, USA) at 37°C for 30 min. After three washes in PBS for 5 min each, the immunoreactive proteins were detected by staining with DAB (Abcam) according to the manufacturer's instructions. Eight magnifications (20×) fields per liver were assessed for the level of p62. The average immunostaining intensities are analyzed.

Primary isolated liver parenchymal and non-parenchymal cells were planted on coverslips for 4 hr (n = 4 per group). These slides were fixed in 4% paraformaldehyde. After 3 washes in PBS for 5 min each, the specimens were covered with 3% BSA at 37°C for 30 min, and then were incubated with primary antibodies at 4°C overnight. These specimens were rinsed in PBS and incubated with Alexa Fluor labeled second antibodies (Invitrogen, CA, USA) at 37°C for 30 min. After three washes in PBS for 5 min each, the slides were covered with DAPI, and detected by confocal microscopy (Leica).

### Apoptosis assay

Single-cell suspensions were prepared from unprocessed normal liver tissues (n = 4 per group), as follows. The liver was minced with scissors into pieces of 1 mm and placed in 10 ml of ice-cold dissociation medium. After draining through a plastic tea sieve, the pieces of tissue were re-suspended in 50 ml of cold, fresh dissociation medium and kept on ice until further processing when the suspensions were placed in an atmosphere of 95% 0_2_ and 5% C0_2_ with continuous shaking at 37°C. After 20 min, the non-dissociated pieces of tissue were collected by passing the suspension through a tea sieve and then re-incubated in 50 ml of fresh dissociation medium. This process was repeated three times. Cells from each filtrate were collected by centrifugation (70×g, 30°C, 10 min), then washed in 10 ml of incubation medium, re-centrifuged, and resuspended in 50 ml of incubation medium to give the working dilution. The percentage of apoptotic cells were assessed by staining with Annexin V-APC and propidium iodide and detection by flow cytometer. For each measurement, >10,000 events were counted.

TUNEL (terminal deoxynucleotidyl transferase–mediated dUTP nick-end labeling) staining was performed with a kit (Roche, USA) following the manufacturer's instructions (n = 4 per group). Eight magnifications (20×) fields per liver were assessed for the level of p62. The percentage of TUNEL-positive cells is analyzed.

### Statistical analysis

All statistical analyses were carried out using the SPSS version 11.5 software (Chicago, IL, USA). Data are expressed as mean ± SD. Between-group differences were analyzed using the two-tailed Student's *t*-test. A *p*-value of <0.05 was considered statistically significant.

## Results

### DEN causes more liver tumors in TLR2-deficient mice and mice treated with anti-TLR2 antibody

**Figure 1:**
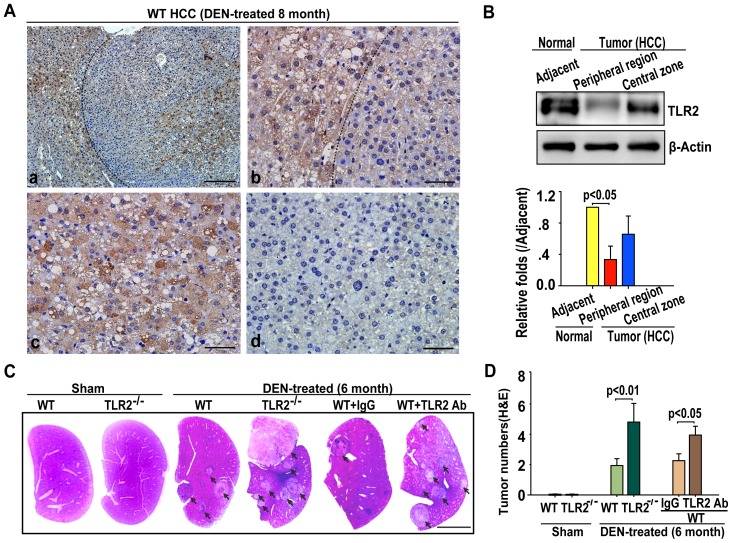
TLR2 deficiency promotes DEN-induced HCC in mice. (A) Representative images of TLR2 immunostaining in liver tissue from WT mice at 8 months post-DEN injection (n = 4 per group). (a) Histological image of HCC with an anti-TLR2 antibody. Scale bars, 200 µm. (b) Histological image of HCC with an anti-TLR2 antibody to show normal tissue adjacent HCC and peripheral region of HCC. Scale bars, 50 µm. (c) Histological image of HCC with an anti-TLR2 antibody to show central zone of HCC. Scale bars, 50 µm. (d) Histological image of negative control with an anti-IgG antibody. Scale bars, 50 µm. (B) Expression of TLR2 was detected in normal tissue adjacent HCC, peripheral region and central zone of HCC. Data are presented immune blots (top panel) and mean ± SEM (n = 4 per group, bottom panel). (C) Representative H&E images of liver sections from the WT, TLR2^−/−^, IgG-treated WT, or WT mice treated with anti-TLR2 at the six-month after DEN treatment. Scale bars, 1 cm. (D) Blocking TLR2 activity, either by TLR2 knockout or by anti-TLR2 antibody, enhanced the numbers of DEN-induced tumor nodules detected by H&E staining (n = 15 per group).

DEN treatment induced liver tumors in all of the WT and TLR2^−/−^ mice within six month of injection. We first examined the expression of TLR2 in liver tissues after HCC occurred. Immunohistochemical analysis showed that the highest expression level of TLR2 was found in normal liver tissue adjacent HCC; TLR2 expressed in the central zone of HCC was higher than that in the HCC peripheral region (Figure 1A). Immune blotting analysis verified that the expression level of TLR2 was lower in the HCC peripheral region than that in HCC adjacent area (Figure 1B). These findings suggested the roles of TLR2 in the development of the DEN-induced HCC. Indeed, a significantly increased number and larger volume of liver tumors had been observed in TLR2^−/−^ mice in compared to WT counterparts at 6 months after DEN injection (Figure 1C, D). Blockade of TLR2 signaling by the pretreatment of WT mice with an anti-TLR2 antibody also increased the number, volume and size of DEN-induced HCCs (Figure 1C, D). These results suggest that the TLR2′s activity can protect against DEN-induced HCC development.

### Enhanced DEN-induced HCC by TLR2-deficiency is associated with ROS accumulation and ER stress

**Figure 2:**
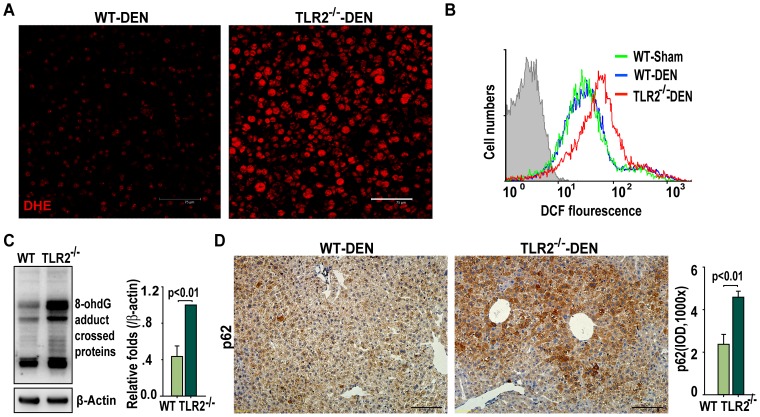
TLR2-deficiency results in increases in ROS accumulation and oxidative DNA damage in DEN-injured liver tissues. (A) Representative images of DHE-detected ROS (left panel, red) in the livers of WT and TLR2^−/−^ mice at one month post-DEN injection (n = 4 per group). Scale bar, 75 µm. (B) DCF fluorescence-detected ROS in livers of WT and TLR2^−/−^ mice (n = 4 per group). (C) Western blot detection of 8-hydroxyguanosine adduct crossed proteins in livers of WT and TLR2^−/−^ mice at one month post-DEN injection. Data are presented as mean ± SEM (n = 4 per group). (D) Representative images of p62 immunostaining in livers of WT and TLR2^−/−^ mice at one month post-DEN injection (left panel). Scale bars, 100 µm. The p62 intensities are presented as mean ± SD (n = 4 per group, right panel).

To test whether the increased tumor number in TLR2^−/−^ mice was associated with the oxidative stress and/or ER stress, we measured the expression and distribution profiles of oxidative stress and ER stress markers. We found that more ROS accumulated in the liver tissues from TLR2^−/−^ mice than WT mice at one month after DEN injection (Figure 2A and 2B). Also, the expression level of 8-hydroxyguanosine (8-ohdG), a biomarker of oxidative stress-damaged products of DNA, increased remarkably in the TLR2^−/−^ mice (Figure 2C).

**Figure 3:**
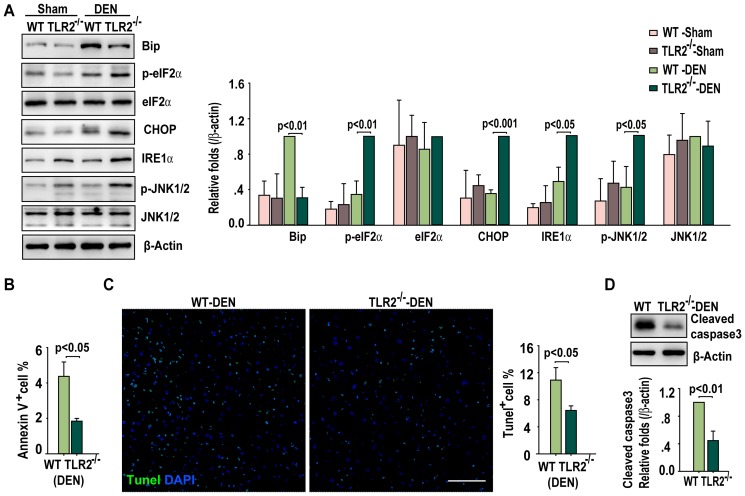
TLR2 deficiency enhances ER stress in DEN-injured liver tissues. (A) Western blot detection of Bip, phospho-eIF2α, eIF2α, CHOP, IRE1α, phospho-JNK1/2 and JNK1/2 in livers of WT and TLR2^−/−^ mice at one month post sham or DEN treatment. Data are presented as mean ± SD (n = 4 per group, right panel). (B) Apoptosis quantified by Annexin V staining is presented as mean ± SD (n = 4 per group). (C) Representative images of TUNEL staining (green) with liver sections from WT and TLR2^−/−^ mice at one month post DEN treatment. The cell nuclei were counterstained with DAPI (blue). Scale bar, 75 µm. Eight magnified (20×) fields per liver were counted for the percentage of TUNEL-positive cells. Data are presented as mean ± SD (n = 4 per group, right panel). (D) Western blot detection of cleaved caspase-3 in livers of WT and TLR2^−/−^ mice at one month post DEN treatment. Data are presented as mean ± SD (n = 4 per group, bottom panel).

Because intracellular accumulation of ROS may either result from the accumulation of p62, a cargo receptor of selective autophagy, or result in the aggregation of p62 in cells [27], [28], we examined the protein level of p62 in the liver tissues obtained from the different groups of mice. The expression of p62 was significantly higher in the livers from the DEN-treated TLR2^−/−^ mice than that from the DEN-treated WT mice (Figure 2D). Western blotting revealed that the DEN-injured liver from the TLR2^−/−^ mice showed an enhanced unfold protein response (UPR) (Figure 3A): the ER stress chaperone protein, Bip, was down-regulated and ER stress sensors such as phospho-eIF2α, IRE1α, and CHOP were up-regulated in TLR2^−/−^ mice, indicating an increase in ER stress in the livers. In contrast to the results of increased ROS accumulation and ER stress, less apoptosis was induced in the DEN-injured TLR2^−/−^ livers than their WT counterparts' livers (Figure 3B to D).

Our previous work indicates that the liver-infiltrating macrophages reduce in TLR2 deficient mice in response to DEN insult [22]. Thus, TLR2 regulation of DEN-induced cancerogenesis is a liver cell specific effect through a cytochrome P450 2E1 dependent ROS-generating manner in liver [32]. Hepatocytes are more susceptible to DEN-induced damage than other types of liver cells. Thus, we evaluated the expression of TLR2 and F4/80 (a marker of marophages) in parenchymal and non-parenchymal cells by Immunofluorescence-staining analysis. We found that not only kupffer cells but also hepatocytes were TLR2 positive (Figure S1A). Indeed, more ROS production and p62 aggregates were detected in parenchymal than non-parenchymal cells (Figure S1B); and treatment of these cells with H_2_O_2_ caused more ROS and p62 accumulation in TLR2^−/−^ parenchymal cells than WT parenchymal cells (Figure S1C). We also found a decrease in the expression of phospho-eIF2α, CHOP, IRE1α, and phospho-JNK in HepG2 cells stably overexpressing TLR2 (Figure S2C). We next examined if TL2 could modulate stresses in other cell types. An increase in the expression of phospho-eIF2α, CHOP, and phospho-JNK was found in primary isolated MEFs from TLR2^−/−^ mice in a manner independent of DEN treatment (Figure S2A). Thus, this modulation seems not limit to liver cells or DEN challenge.

### Antioxidant reagent NAC attenuates DEN-induced HCC in TLR2-deficient mice

**Figure 4:**
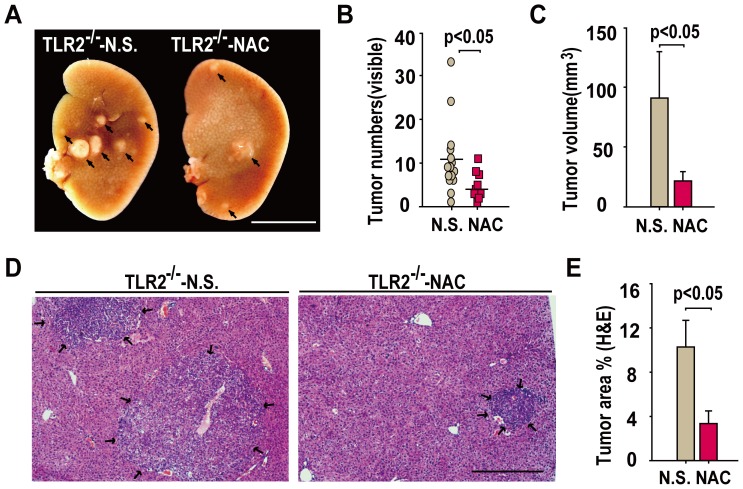
Antioxidant NAC ameliorates TLR2^−/−^-aggravated HCC progression. (A) Representative images of gross liver specimens from TLR2^−/−^ mice treated with or without NAC at six months post-DEN injection. Scale bars, 1 cm. (B) NAC treatment decreased the number of surface tumor nodules in TLR2^−/−^ mice. Each dot represents the tumor nodules from each mouse. Scale bars indicate the mean ± SD (n = 20 per group). (C) NAC treatment decreased the volume of surface tumors. Data are presented as mean ± SD (n = 20 per group). (D) Representative H&E stained liver sections from TLR2^−/−^ mice treated with or without NAC. Scale bar, 500 µm. (E) The calculated tumor area (percentage of liver) is presented as mean ± SD (n = 20 per group).

Because TLR2 deficiency aggravated HCC associated with oxidative and ER stress, we tested whether an antioxidant agent had a protective role in the DEN-induced HCC in TLR2^−/−^ mice. Indeed, NAC treatment led to significantly less numbers, smaller volume and sizes of HCC in the DEN-injured liver from TLR2^−/−^ mice when compared to the untreated TLR2^−/−^ mice (Figure 4A–E). These observations indicate that the anti-hepatocarcinogenesis effect of TLR2 signaling in response to DEN insult, at least partially, relies on the role of TLR2's activity in the attenuation of ROS accumulation and oxidant stress.

**Figure 5:**
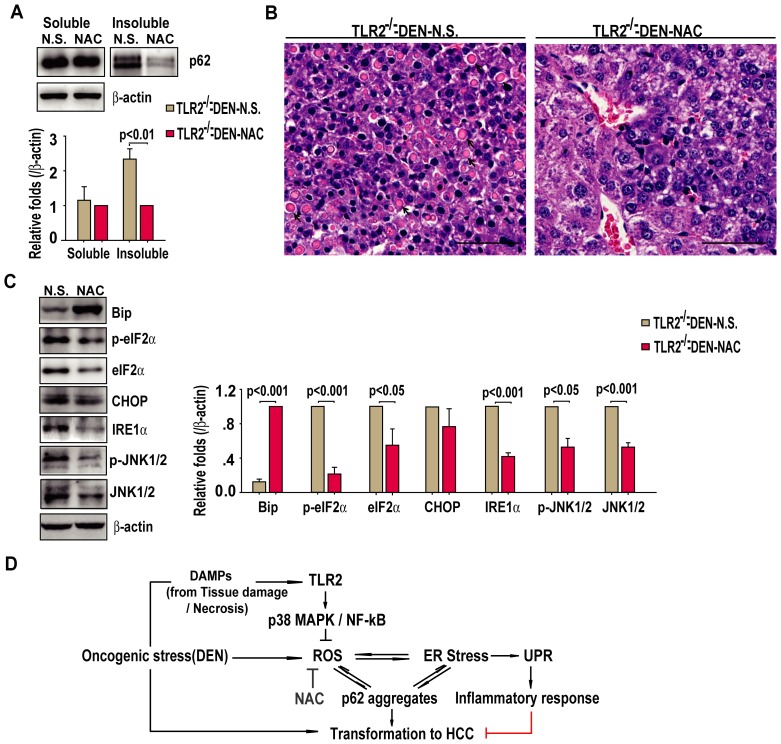
Antioxidant NAC ameliorates TLR2^−/−^- exacerbated ER stress in HCC tumors. (A) Western blot detection of p62 in the detergent-soluble and insoluble fractions of liver tissues of DEN-induced TLR2^−/−^ mice treated and untreated with NAC. Data are mean ± SEM (n = 4 per group, bottom panel). (B) Representative images of H&E stained liver showing Mallory-Denk bodies (arrow, n = 4 livers per group). Scale bar, 50 µm. (C) Western blot detection of Bip, phospho-eIF2α, eIF2α, CHOP, IRE1α, phospho-JNK1/2 and JNK1/2 in livers tissues of DEN-induced TLR2^−/−^ mice treated and untreated with NAC. Data are mean ± SEM (n = 4 per group, right panel). (D) A schematic diagram showing how TLR2 may protects against DEN-induced hepatocarcinogenesis. Upon exposure to oncogenic stressors, such as DEN, TLR2-mediated signaling prevents ROS accumulation and ER stress, thereby preventing p62 aggregation and discouraging transformation. Inhibition of TLR2 signaling in liver under the conditions of oncogenic stressors could lead to uncontrolled ROS accumulation and ER stress, promoting tumorigenesis of liver cells.

Because NAC treatment was able to reduce the DEN-induced HCC development in TLR2^−/−^ mice, we tested whether this agent inhibited the accompanying increase in ER stress factors. We found that the treatment of mice with NAC reduced the levels of insoluble p62 aggregates, but had little effect on p62 in the soluble fraction in liver (Figure 5A). The livers from NAC-treated TLR2^−/−^ mice also showed a significantly reduced number of Mallory-Denk bodies, which are cytoplasmic protein aggregates in hepatocytes (Figure 5B). Consistent with these findings, NAC treatment was also shown to significantly up-regulate Bip and down-regulate phospho-eIF2α, phospho-JNK1/2, total eIF2α, JNK1/2, and IRE1α(Figure 5C). Intriguingly, NAC treatment had no detectable effect on the ER stress sensor CHOP (Figure 5C). Collectively, these results suggest that TLR2 activity prevents the DEN-induced HCC by diminishing oxidative stress and ER stress, which induces autophagic flux and attenuates UPR-mediated inflammation in response to DEN liver injury (Figure 5D).

## Discussion

HCC has been characterized as a chronic inflammation-driven cancer and the studies of animal models of chemically-induced HCC have revealed the crucial roles of inflammatory signaling in disease onset and severity [33]. Hepatic immunity is predominantly innate [16]. The pattern recognition receptors, especially TLRs, play a central role in maintaining such balance between homeostasis and inflammatory state in liver [34]. We recently find that knockout TLR2 enhances DEN-induced hepatocarcinogenesis because the liver immune network fails to respond to DEN challenge in TLR2 deficient livers, resulting in a less infiltration of macrophages and release of immune factors. This immune-network is necessary for maintaining the functional autophagy flux, cellular senescence, and cellular death undergoing cellular stress [22], [23]. In this study, we further prove that ROS/ER stress is directly responsible for the aggravation of liver carcinogenesis in TLR2 deficient mice. The treatment of animals with the antioxidant agent NAC can attenuate ROS/ER stress to prevent UPR-induced inflammation and p62 aggregation in DEN-treated TLR2^−/−^ liver. Thus, NAC treatment interrupts the positive feedback of the ROS/ER stress-p62 aggregation-UPR-induced inflammation and reverses the TLR2 deficiency increased susceptibility of HCC development and progression.

In hepatocytes, DEN is metabolized by cytochrome P450 2E1 via a ROS-generating reaction. DEN administration thus causes a large amount of ROS production in WT hepatocytes, which is not only responsible for liver injury and necrosis of hepatocytes, but also induces DNA damage and genomic instability [34]. Consequently, DAMPs are released from these injured and necrotic hepatocytes and activate PRRs on macrophages and liver cells. The activated macrophages intake debris of necrotic hepatocytes and secrete immune factors that further activate autophagy and senescence response in hepatocytes [22]. The activated immune network in turn supports the cell death and survival signaling pathways of hepatocytes to counter against cellular stresses. Therefore, the production of ROS plays a critical role in maintaining homeostasis in response to DEN insult to protect against tumorigenesis and progression.

ER stress is triggered when unfolded proteins accumulate in the ER and is one of the endogenous sources of ROS accumulation [26]. P62 is a cargo receptor of selective autophagy and can deliver ubiquitin decorated proteins into the autophagy-lysosome degradation pathway [35]. Once cells are undergoing ER stress or failure of autophagic flux, p62 and its bonded proteins would became aggregation. P62 aggregation is another source of ROS production and accumulation [28]. In turn, ROS is a powerful trigger of ER stress and p62 aggregates [26], [28]. Thus, a positive feedback loop is established among the ROS production, ER stress and p62 aggregation in DEN-injured liver. For this reason, once the accumulated ROS is reduced by the treatment of NAC, the ER stress and p62 aggregates are decreased and HCC development is diminished in the TLR2^−/−^ liver.

Activation of TLR2 stimulates several signaling pathways in response to the DEN-induced ROS accumulation. The ASK1/p38 MAPK/NF-κB signaling pathway is a major sensor for cellular ROS and drives these higher mutant risk cells into apoptotic cell death [29]. Each of the ERK, JNK, and Akt pathways has been reported involving in the positive or negative regulation of proliferation, apoptosis, and autophagy in response to ROS. For instance, The UPR-activated JNK pathway links the ER-stress to inflammation in much broader cell types in responding to cellular stress [26]. The activation of autophagy under ER-stress is necessary for cell survival in a JNK dependent manner [36]. Therefore, JNK is an important downstream molecule of UPR and plays a dominant pro-survival role through an UPR-JNK-autophagy pathway [37]. Indeed, DEN treatment causes an attenuation of the ASK1/p38 MAPK/NF-κB and PI3K/Akt signaling [22], [23] but an increase in the activity of JNK pathways (Figure 3A) in livers from TLR2-deficient mice, suggesting that HCC cells containing higher ROS and DNA damages have more chance to survive in TLR2^−/−^ livers.

Molecular therapeutic strategies against cancer by targeting the components of the innate immune system rely on a precise understanding of the tumor stage and pathogenic mechanisms [38]. TLR agonists have been shown to enhance immune responses against tumors in both animal models and humans. Indeed, the anti-tumor activities of Imiquimod, a ligand for TLR7, and CpG DNA, a ligand for TLR9, have been demonstrated in several clinical trials [39], [40]. TLR2 agonist MALP-2, a 2 kDa synthetic lipopeptide with macrophage-stimulatory activity, is currently in use as a cancer immunotherapy [41], [42]. Based on the evidence presented herein, which shows that TLR2 prevents HCC development, it is possible that MALP-2 may be a useful activator of TLR2 for suppressing HCC development.

In conclusion, a molecular mechanism underlying the TLR2-mediated anti-tumor effects involves, at least partially, the prevention of ROS accumulation and ER stress onset. More importantly, treatment with the antioxidant reagent, NAC, can eliminate ROS accumulation, alleviate the ER stress, interrupt the positive feedback of p62 aggregation and ROS production in the DEN-injured liver tissue, and reduce the burden of liver tumors. Subsequent studies are needed to uncover the precise components and signal pathway and their dynamic interactions that regulate the signal from TLR2 activation to suppression of oxidant and ER stressors in HCC.

## Supporting Information

Figure S1 **Expression of TLR2 associates with ROS accumulation and p62 aggregates in parenchymal cells.** (A) Representative images of WT primary parenchymal (left panel), non-parenchymal (middle panel), and TLR2^−/−^ non-parenchymal cells (right panel) stained with TLR2 and F4/80. Scale bar, 10 µm. (B) Liver parenchymal cells produced more ROS and p62 aggregates. Primary parenchymal cells (PC, red, left panel) and non-parenchymal cells (NPC, black, middle panel) was detected by flow cytometer, and measured for ROS and p62 aggregates (right panel). (C) H_2_O_2_ induced more ROS and p62 aggregates in TLR2^−/−^ parenchymal cells. Primary isolated WT and TLR2^−/−^ parenchymal cells was treated with 50 µM H_2_O_2_ for 2 hrs, and was detected by flow cytometer (PC, blue, left panel). WT (red) and TLR2^−/−^ (black) parenchymal cells was measured for ROS and p62 aggregates (middle panel). H_2_O_2_ treatment induced more ROS and p62 aggregates in TLR2^−/−^ parenchymal cells (n = 4, right panel).
(TIF)

Figure S2 **Expression of TLR2 associates negatively with ER stress in MEFs or HepG2 cells.** (A) Expression of Bip, phospho-eIF2α, eIF2α, CHOP, IRE1α, phospho-JNK1/2 and JNK1/2 was detected with Western blotting in MEFs treated with or without DEN (200 µg/ml for 24 h). Data are representative immune blots (left panel) and mean ± SEM (n = 4 per group, right panel). (B) The stable overexpression of TLR2 was identified in the clone 1 of HepG2 cells. (C) Expression of Bip, phospho-eIF2α, eIF2α, CHOP, IRE1α, phospho-JNK1/2 and JNK1/2 was detected with Western blotting in HepG2 cells over-expressing TLR2 or control vector. Data are representative blots (left panel) and mean ± SEM (n = 4 per group, right panel).
(TIF)
