# Supplementary figures and images for: Antioxidant N-Acetylcysteine Attenuates Hepatocarcinogenesis by Inhibiting ROS/ER Stress in TLR2 Deficient Mouse
Source: PLoS One. 2013 Oct 2;8(10):e74130. doi: 10.1371/journal.pone.0074130 (PMC3788783; doi:10.1371/journal.pone.0074130)

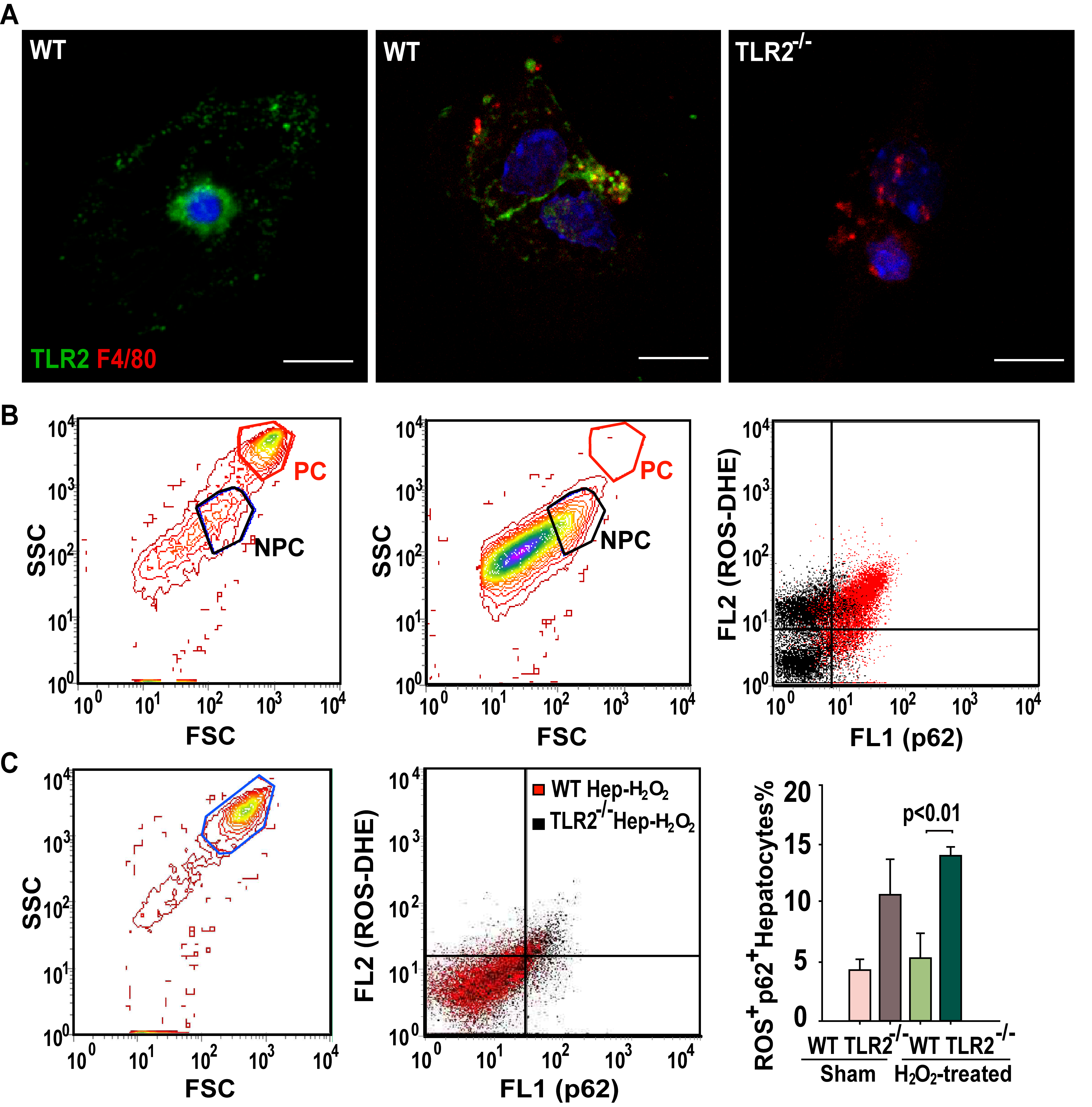

Supplement: Figure S1 — Expression of TLR2 associates with ROS accumulation and p62 aggregates in parenchymal cells. (A) Representative images of WT primary parenchymal (left panel), non-parenchymal (middle panel), and TLR2−/− non-parenchymal cells (right panel) stained with TLR2 and F4/80. Scale bar, 10 µm. (B) Liver parenchymal cells produced more ROS and p62 aggregates. Primary parenchymal cells (PC, red, left panel) and non-parenchymal cells (NPC, black, middle panel) was detected by flow cytometer, and measured for ROS and p62 aggregates (right panel). (C) H2O2 induced more ROS and p62 aggregates in TLR2−/− parenchymal cells. Primary isolated WT and TLR2−/− parenchymal cells was treated with 50 µM H2O2 for 2 hrs, and was detected by flow cytometer (PC, blue, left panel). WT (red) and TLR2−/− (black) parenchymal cells was measured for ROS and p62 aggregates (middle panel). H2O2 treatment induced more ROS and p62 aggregates in TLR2−/− parenchymal cells (n = 4, right panel). (TIF) [file pone.0074130.s001.tif]

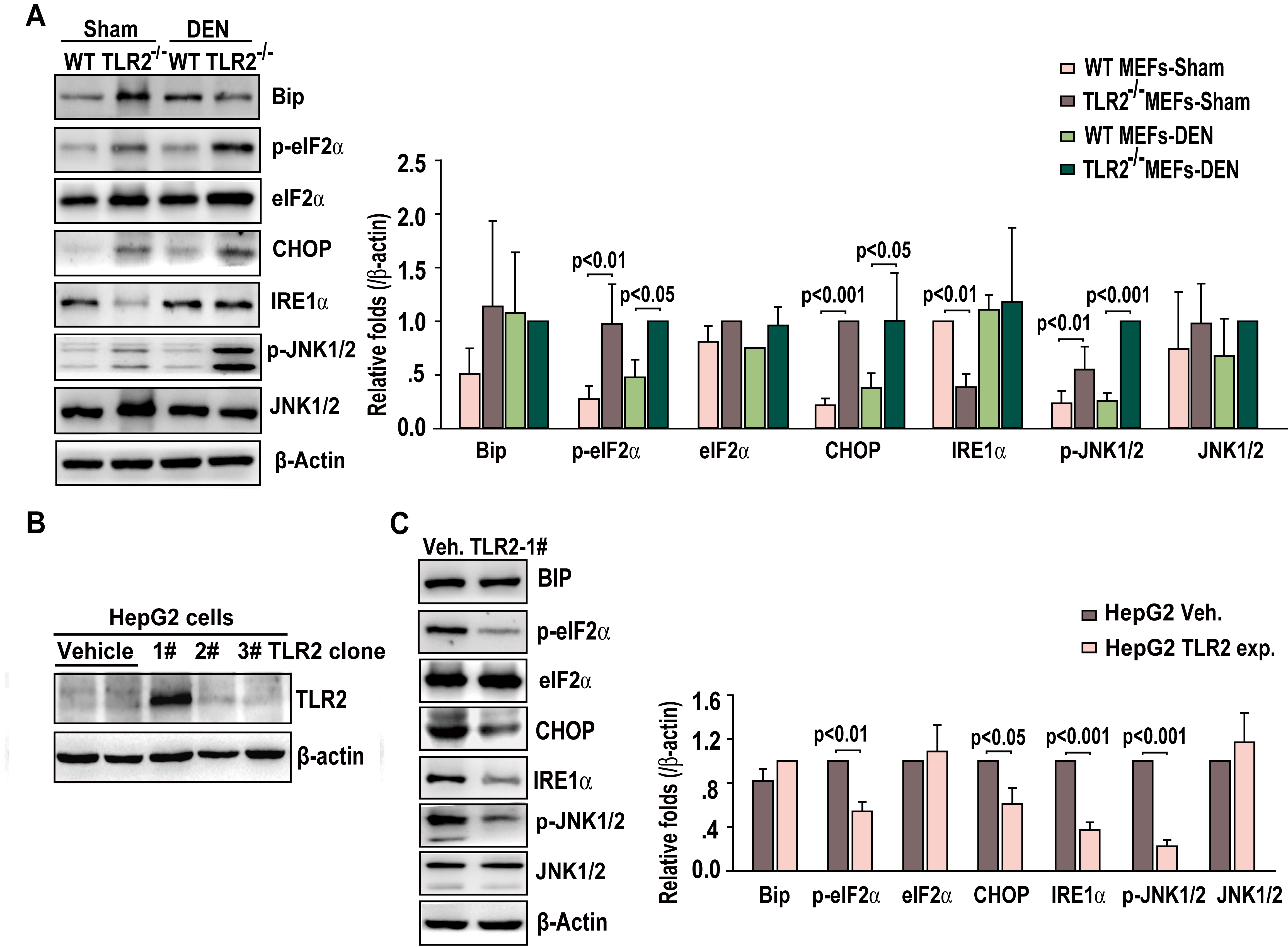

Supplement: Figure S2 — Expression of TLR2 associates negatively with ER stress in MEFs or HepG2 cells. (A) Expression of Bip, phospho-eIF2α, eIF2α, CHOP, IRE1α, phospho-JNK1/2 and JNK1/2 was detected with Western blotting in MEFs treated with or without DEN (200 µg/ml for 24 h). Data are representative immune blots (left panel) and mean ± SEM (n = 4 per group, right panel). (B) The stable overexpression of TLR2 was identified in the clone 1 of HepG2 cells. (C) Expression of Bip, phospho-eIF2α, eIF2α, CHOP, IRE1α, phospho-JNK1/2 and JNK1/2 was detected with Western blotting in HepG2 cells over-expressing TLR2 or control vector. Data are representative blots (left panel) and mean ± SEM (n = 4 per group, right panel). (TIF) [file pone.0074130.s002.tif]
